# Recently resettled refugee women-at-risk in Australia evidence high levels of psychiatric symptoms: individual, trauma and post-migration factors predict outcomes

**DOI:** 10.1186/s12916-018-1143-2

**Published:** 2018-09-18

**Authors:** Robert D. Schweitzer, Lyn Vromans, Mark Brough, Mary Asic-Kobe, Ignacio Correa-Velez, Kate Murray, Caroline Lenette

**Affiliations:** 1 School of Psychology and Counselling, Queensland University of Technology, Kelvin Grove, QLD 4067 Australia; 2 School of Public Health and Social Work, Queensland University of Technology, Kelvin Grove, QLD 4067 Australia; 3 Access Community Services Ltd, Logan Central, QLD 4114 Australia; 4 Forced Migration Research Network, School of Social Sciences, University of New South Wales, Sydney, NSW 2006 Australia

**Keywords:** Refugee, Women, Mental health, Trauma, Women-at-risk, Post-migration living difficulties

## Abstract

**Background:**

Despite increasing numbers of refugee women-at-risk being resettled and their potential vulnerability, there exists no empirical research into the psychiatric health of this unique subgroup with which to guide policy and practice. This research aimed to investigate psychiatric symptom status of a sample of refugee women-at-risk recently resettled in Australia, as well as factors contributing to symptoms of trauma, anxiety, depression, and somatization. The level of psychiatric symptomatology is compared to reference groups of women from Sudan and Burma, who entered Australia under the Humanitarian Entry Programme, and who did not meet criteria as women-at-risk.

**Methods:**

This is a cross-sectional survey of 104 refugee women-at-risk across several ethnic groups including a demographic questionnaire, the Harvard Trauma Questionnaire, Post-migration Living Difficulties Checklist, and Hopkins Symptom Checklist to assess individual factors, traumatic experiences, post-migration problems, and symptoms of trauma, anxiety, depression, and somatization. A series of multiple hierarchical regression analyses examined factors predicting psychiatric symptoms.

**Results:**

Substantial proportions of participants reported psychiatric distress in symptomatic ranges, including for traumatization (41%), post-traumatic stress disorder (20%), anxiety (29%), and depression (41%), as well as significant symptoms of somatization (41%). These findings are significantly higher than those derived from reference groups of women from Sudan or Burma, resettled in the same area and utilizing a similar methodology. Higher numbers of trauma events and post-migration living difficulties predicted higher trauma, depression, and somatic (but not anxiety) symptoms. Having children predicted higher trauma, anxiety, and somatic symptoms. Greater English fluency predicted higher anxiety symptoms. Region of birth predicted anxiety and depression symptoms. Age predicted trauma and anxiety symptoms.

**Conclusions:**

Findings suggest that recently arrived refugee women-at-risk are at high risk of psychiatric disorders. The results indicate a need for comprehensive psychiatric assessment to identify women in need of treatment very early after resettlement, with implications for medical practice, service delivery, and policy programs.

## Background

The United Nations High Commissioner for Refugees (UNHCR) considers that “*those women or girls who have protection problems particular to their gender, and lack effective protection normally provided by male family members*” ([1], p. 263) are vulnerable to gender-related human rights violations in addition to traumas often reported by other refugee groups. The vulnerability of these women and girls means their experience of the refugee journey will likely differ from that of their male or not at-risk female counterparts [1]. Consequently, the UNHCR created the ‘Women-at-Risk’ visa category to expedite suitable protection and support for identified refugee women through resettlement [2]. Applications for this visa are likely to rise since global numbers of forcibly displaced people appear to be growing, reaching 65.6 million by the end of 2016, representing an increase of 300,000 people over 1 year [3]. Increased numbers of forcibly displaced people underscore rises in UNHCR resettlement submissions, which increased from 92,915 in 2013 to 162,575 in 2016 [4]. Women-at-risk have previously comprised approximately 10% of UNHCR submissions for resettlement in countries like Australia, Canada, and the United States [5].

Responding to the growing numbers of forcibly displaced people, the number of countries offering resettlement has also grown, rising from 14 in 2005 to 37 in 2016 [4]. Resettlement countries often work with non-governmental organizations to assess the needs of newly arrived refugees, including their psychiatric needs, and then provide appropriate resettlement support, services, and referrals aimed towards facilitating refugee’s wellbeing and local integration [6]. Consistent with UNHCR global resettlement priorities [1], Australia gives high priority to women-at-risk and their dependents. Australia’s Humanitarian Entry Programme [7] provided 1009 Woman-at-Risk visas (subclass 204) in 2014–2015, representing 7% of the 13,756 Humanitarian visas granted that year. Women-at-risk occasionally enter Australia on other humanitarian visas, such as on Refugee (subclass 200) or Global Special Humanitarian (subclass 202) visas. Empirical investigation of the psychiatric symptoms experienced by women-at-risk is required to assist policy-makers and practitioners to respond effectively to this vulnerable group.

Research-informing policy and practice relating to refugee resettlement has often focused on trauma [8, 9]. Trauma symptoms are the likely sequelae to traumatic events experienced by many refugees, with a proposed dose response [10, 11]. Symptoms sometimes meet criteria for post-traumatic stress disorder (PTSD). For example, research with 63 Sudanese [12] and 70 Burmese refugees [13] found that the number of trauma events experienced by participants predicted their trauma symptoms. However, similarly to many studies in the field, samples included males and females and the gendered nature of the refugee journey was not fully addressed. The experiences of refugee women-at-risk, who are without male protection, are likely to be qualitatively different from other refugee groups, often involving gender-related violence such as rape or sexual bartering [14–16]. Such gender-based assaults are likely to have additional physical and social ramifications, including pregnancies and community ostracism [17, 18].

Post-migration stressors, such as cultural loss [9], communication issues [19], social support [20], racial discrimination [21], and employment [22], can significantly impact mental health. Although meta-analytic research suggests worse outcomes for those accommodated in institutions or with restricted economic opportunities [23], findings on the most serious stressors and their impacts vary across different refugee populations [9, 12, 13]. Both structural and individual factors may influence the kinds of difficulties experienced by distinct refugee groups, as well as consequent impacts on their mental health. When framing the stressors faced by women-at-risk, it is necessary to engage with a wider picture of global patriarchy and challenge assumptions that oppressive circumstances are resolved in resettlement. Indeed, qualitative studies suggest that women-at-risk often remain vulnerable to violence and exploitation after resettlement [17]. The wellbeing of refugee women-at-risk is best understood in a way that is mindful of the diversity of women’s experiences and recognizes potential for ongoing oppressions that women may face in resettlement countries.

Determining potential contributing factors specific to the mental health of women-at-risk has utility in informing future policy, assessment, and practice protocols for this group. Meta-analytic research with the wider refugee population has found that being female, older, and more educated are each associated with poorer mental health [23]. Having children, with the concomitant tasks and responsibilities of lone parenting can be overwhelming, as reported by resettled Congolese women with children in the United States [16]. With migration increasingly viewed through the spectre of terrorism, potential difficulties from religious affiliation are highlighted by the fear for personal, psychological, and cultural insecurity reported by Muslim refugee women [21]. Research with South-East Asian refugees in Canada indicated that English ability predicted depression in the longer term, but not during the initial period of resettlement [24]. Refugee women-at-risk comprise a demographically heterogeneous group and individual characteristics may impact their resettlement experiences and wellbeing.

While previous research points to the vulnerability of refugee women-at-risk both before [2] and after [17] migration, to our knowledge, the psychiatric symptom status of recently resettled refugee women-at-risk has not been documented. Further, there exists no empirical research examining contributions of individual factors, traumatic events, or post-migration problems to their psychiatric symptoms. Given that numbers of resettled refugee women-at-risk are likely to rise, research that assists our understanding of risk factors associated with their mental health has the potential to inform policy in resettlement countries and guide practitioner assessment and practice protocols so that we might better respond to their unique needs.

## Methods

### Aims

The current study sought to (1) determine the prevalence of psychiatric symptoms (trauma, anxiety, depression, and somatic) in a sample of refugee women-at-risk, recently resettled in Australia; (2) index the traumatic events and post-migration difficulties experienced by these women; (3) compare levels of psychiatric symptomatology to reference groups of women from Sudan and Burma, who entered Australia under a Humanitarian Entry Programme, and who did not meet criteria as women-at-risk; and (4) examine contributions of individual factors, traumatic events, and post-migration living difficulties to the women’s psychiatric symptom levels. Based on previous, repeated associations found between trauma experiences and trauma symptoms [10], we expected that a greater number of trauma events would predict higher levels of trauma symptoms. Given previous research findings [9, 12, 13], we expected post-migration living difficulties to predict higher psychiatric symptom levels. Given previous meta-analytic findings [23], we expected that higher age and education would predict higher psychiatric symptom levels.

### Study population

Participants were 104 women from refugee backgrounds, who (1) were aged over 18 years old; (2) entered Australia within the previous 6 months; (3) were referred to the research as women-at-risk by a resettlement agency; and (4) provided voluntary informed consent to participate in the research. Eighty-eight of the women entered Australia under the Women-at-Risk visa category (visa subclass 204) with the remaining 16 women entering under the more general Humanitarian visa category (visa 200). However, all participants were assessed as meeting the definition of women-at-risk. Women were referred by a non-governmental agency consecutively to their arrival in Australia, and according to their willingness to participate in the study. The findings are designed to be generalizable to women-at-risk, rather than to people from refugee populations as a whole. The agency, funded by the Australian Department of Social Services to provide settlement support services to newly arrived refugees (ACCESS Community Services), assisted recruitment from 2013 to 2015.

### Measures

#### Demographic questionnaire

A demographic questionnaire enquired into age, country of birth, visa category, religion, marital status, number of children, educational level, and English skills.

#### Harvard Trauma Questionnaire (HTQ)

The HTQ [25] is widely used in refugee research [26, 27], measuring trauma experiences and symptoms. Part 1 comprised 17 items measuring participants’ experiencing and witnessing of 17 human rights violations. Part 2 comprised 16 items enquiring into the extent that participants experienced 16 PTSD symptoms. Items were scored on a 4-point ordinal severity scale including not at all (1), a little (2), quite a bit (3), and extremely (4). Higher scores indicated higher distress. In the current study, the HTQ symptom scale had good internal consistency, with a Cronbach’s alpha of 0.86.

#### Hopkins Symptom Checklist (HSCL-37)

The HSCL-37 is a self-report inventory, which extends the HSCL-25 [28] to measure anxiety (10 items), depression (15 items), and somatization (12 items) symptoms [12, 13]. Items were scored on a 4-point ordinal severity scale including not at all (1), a little (2), quite a bit (3), and extremely (4). Higher scores indicated higher distress. The HSCL-37 has demonstrated good reliability with refugee populations [12, 13]. Cronbach alphas in the current study revealed good internal consistency for anxiety (α = 0.86), depression (α = 0.85), and somatization (α = 0.79) subscales.

#### Post-migration Living Difficulties Checklist (PMLD)

The PMLD assessed post-migration stressors experienced by participants. To decrease the load on participants, the PMLD in the current study was adapted from the original 23-item PMLD [29] to include only 10 of the most relevant items to index communication difficulties, discrimination, worry about family members overseas, employment, immigration processes, access to health and welfare services, adjustment to life in Australia, transport, loneliness and boredom, and isolation. Participants indicated whether each problem was not a problem (0), a small problem (1), a moderately serious problem (2), a serious problem (3), or still a problem today (4). Adapted versions of the checklist have been used previously in refugee research [9, 12, 13]. Each item measured a different post-migration experience, making reliability irrelevant.

### Procedure

This cross-sectional survey research was part of a larger longitudinal study approved by the Queensland University of Technology Human Research Ethics Committee (1400000141). Assisted by the resettlement agency, researchers provided women from the target population in South-East Queensland, Australia, with research information and invitation to participate across 2013 (3.9%), 2014 (58.7%), and 2015 (37.5%). Women who provided informed consent were administered the survey battery (approximately 2 hours) at the agency office or in their home, according to participant preference. Researchers prioritized participant welfare throughout and optimized communication and cultural appropriateness by working with referral agency bicultural workers. Bicultural workers received in-house training and supervision in working ethically and effectively with practitioners in assessment of people from refugee backgrounds and spoke the participants’ preferred language.

### Statistical analyses

Following preliminary descriptive and assumption analyses, a series of hierarchical multiple regression analyses examined contributions of selected individual factors, number of trauma events experienced, and level of post-migration living difficulties to participants’ psychiatric symptom levels (trauma, anxiety, depression, and somatic), using a significance criterion of 0.05. Separate hierarchical multiple regression analyses were conducted to assess contributions of the most prevalent PMLD items. Analyses employed SPSS EXPLORE and REGRESSION.

Data from two previously published research studies [12, 13] are presented in the Results section to assist clarity in the later Discussion and are not intended to represent formal comparison groups. Subsets of research participants from those prior studies included Sudanese (*n* = 15) and Burmese (*n* = 34) women, respectively, resettled in Australia on refugee visa categories other than Women-at-Risk visas. Both studies used research protocols similar with the current research, including recruitment strategies and assessments. The age of women in the Sudanese and Burmese groups and their education profiles were broadly similar to those of the current sample. However, religious, marital, and parent profiles of the groups differed from the current sample. Sudanese women were largely Christian (93%), married (80%), with one or more children (87%). Burmese women were either Christian (79%) or Buddhist (21%), with the majority being married (53%) with one or more children (82%). Similar to the current sample, Burmese women had been resettled for a short time (*M* =4.18 months, *SD* =4.51, range 2–25). Although data was not available to calculate average time since resettlement for the Sudanese sample, the larger research population, including all males and females, had been resettled in Australia for just under 2 years on average; longer than the current sample [12]. Please refer to publications for further research descriptions [12, 13].

## Results

### Participant characteristics

Table 1 shows descriptive statistics for participant demographic characteristics. The majority of participants entered Australia on Women-at-Risk visas, many with children. A large proportion of women came from African (*n* = 82; 78.9%) countries (including Eritrea, Democratic Republic of Congo, Ethiopia, Sudan, South Sudan, Rwanda, Burundi, and Kenya), with other women (*n* = 22; 21.1%) coming from countries within South Asia (Afghanistan), West Asia (including Iran, Iraq, and Syria), and South-East Asia (including Myanmar and Thailand). The majority of women were Christian. Most women (*n* = 82; 78.8%) reported being without a male partner (single, divorced, separated, or widowed), with 22 (21.1%) reporting being married or in a de facto relationship. While many women (*n* = 54; 51.9%) had primary or no education, 50 (48.1%) had secondary, trade, or university education.

**Table 1:** Participant demographic characteristics (*N =* 104) and comparison with Sudanese^a^ (*n* = 15) and Burmese^b^ (*n* = 34) women who arrived through Humanitarian (201) or Refugee (subclass 200) visas

Variable	Women-at-risk	Sudanese^a^	Burmese^b^
Age, mean ± SD (range)	32.5 ± 11.6 (18–70)	31.3 ± 5.5 (21–41)	32.7 ± 13.7 (18–79)
Months in Australia, mean ± SD (range)	2.9 ± 1.9 (0–7)	< 24 months	4.2 ± 4.5 (2–25)
Number of children, mean ± SD (range)	2.2 ± 2.5 (0–13)	3.14 ± 1.79 (0–7)	2.38 ± 1.44 (0–4)
Women with no children, *n* (%)	41 (39.4)	1 (7.1)	6 (17.6)
Women with one child or more, *n* (%)	63 (60.6)	13 (87)	28 (82.4)
Visa			
204 – Woman-at-risk, *n* (%)	88 (84.6)	0	0
201 – Humanitarian or 200 – Refugee, *n* (%)	16 (15.4)	15 (100)	32 (100)
Region of birth, *n* (%)			
Africa^c^	82 (78.9)	15 (100)	0
South Asia^d^	12 (11.5)	0	0
West Asia^e^	7 (6.7)	0	0
South East Asia^f^	3 (2.9)	0	34 (100)
Religion, *n* (%)			
Christian	63 (60.6)	14 (93.3)	27 (79.4)
Muslim	41 (39.4)	1 (6.7)	0
Buddhist	0	0	7 (20.6)
Marital status, *n* (%)			
Married/de facto	22 (21.2)	12 (80)	18 (52.9)
Single	51 (49)	1 (6.7)	13 (38.2)
Widowed	21 (20.2)	0	3 (8.8)
Divorced/separated	10 (9.6)	2 (13.3)	0
Education, *n* (%)			
None	24 (23.1)	3 (20)	3 (8.8)
Primary	30 (28.8)	4 (26.7)	16 (47.1)
Secondary	44 (42.3)	7 (46.7)	15 (44.1)
University	4 (3.8)	1 (6.7)	0
Trade	2 (1.9)	0	0
English skills, *n* (%)			
No skills/great difficulty	64 (61.5)	7 (46.7)	–
Some difficulty/fluent	40 (38.5)	8 (53.3)	–

^a^Using split-group analysis of data from Schweitzer et al. [12]

^b^Using split-group analysis of data from Schweitzer et al. [13]

^c^Including Eritrea, Democratic Republic of Congo, Ethiopia, Sudan, South Sudan, Rwanda, Burundi, and Kenya

^d^Afghanistan

^e^Including Iran, Iraq, and Syria

^f^Including Myanmar and Thailand

### Trauma events experienced

Table 2 shows the range of traumatic events reported by women-at-risk participants. The mean number of traumatic events experienced by women-at-risk was 7.23 (*SD* = 4.12; Range = 0–15), and the most frequently endorsed traumas were lack of food or water, lack of shelter, ill health without medical care, and forced separation from family members. Table 2 also shows the traumatic events reported by Sudanese and Burmese refugee women, who entered Australia through other visa categories, using re-analyzed data from previously published research [12, 13]. Women-at-risk experienced a greater number of traumas compared to Sudanese (*M* = 6.07, *SD* = 4.11, range 0–13) and Burmese (*M* = 5.19, *SD* = 3.06, range 0–13) groups.

**Table 2:** Frequencies and proportion of women-at-risk participants experiencing traumatic events (*N* = 104): comparison with Sudanese^a^ (*n* = 15) and Burmese^b^ (*n* = 32) women who arrived through Humanitarian (201) or Refugee (subclass 200) visas

Item	Trauma event	Women-at-risk		Sudanese^a^	Burmese^b^
		*n*	*%*	*%*	*%*
1	Lack of food or water	66	63.5	73.3	71.9
3	Lack of shelter	61	58.7	66.7	71.9
2	Ill health without access to medical care	56	53.8	26.7	43.8
11	Forced separation from family members	56	53.8	93.3	46.9
10	Being close to death	52	50.0	40.0	37.5
5	Serious injury	50	48.1	20.0	15.6
13	Unnatural death of family or friend	47	45.2	20.0	34.4
16	Torture	46	44.2	26.7	12.5
4	Imprisonment/detention	45	43.3	13.3	12.5
12	Murder of family or friends	43	41.3	80.0	21.9
6	Combat situation	42	40.4	46.7	53.1
15	Lost or kidnapped	42	40.4	13.3	9.4
9	Forced isolation from others	37	35.6	33.3	9.4
8	Rape or sexual abuse^c^	34	33.7	13.3	16.1
14	Murder of stranger or strangers	34	32.7	26.7	15.6
7	Brain washing	25	24.0	13.3	21.9
17	Threatened by dangerous animals	16	15.4	00.0	25.0

^a^Using split-group analysis of data from Schweitzer et al. [12]

^b^Using split-group analysis of data from Schweitzer et al. [13]

^c^Three participants chose not to answer this question in the women-at-risk group

### Post-migration living difficulties

Table 3 shows the proportion of participants reporting selected post-migration living difficulties as serious problems. Items most frequently endorsed by women-at-risk as having been serious problems or still problems in their lives included worry about family overseas, communication, loneliness and boredom, and transport. A high proportion of participants reported experiencing no difficulties with racial discrimination, adjusting to cultural life in Australia, or employment. Table 3 also shows post-migration living difficulties experienced by the Sudanese and Burmese groups [12, 13].

**Table 3:** Proportion of women-at-risk reporting post-migration living difficulties as serious or current problems (*N* = 104): comparison with Sudanese^a^ (*n* = 14) and Burmese^b^ (*n* = 32) women who arrived through Humanitarian (201) or Refugee (subclass 200) visas

Item	Difficulty	Women-at-risk	Sudanese^a^	Burmese^b^
3	Worry about family not in Australia	79.8	92.9	59.4
1	Communication	40.4	42.8	62.5
9	Loneliness and boredom	28.8	–^c^	–^c^
8	Transport	24.0	–^c^	–^c^
4	Employment	17.3	57.1	9.4
10	Feeling isolated from other people	16.4	–^c^	–^c^
5	Immigration/asylum Processes	16.3	7.1	3.1
6	Accessing health and welfare services	11.6	0.0	9.4
2	Racial discrimination	2.0	7.1	0
7	Adjusting to cultural life in Australia	7.7	14.2	0

^a^Using split-group analysis of data from Schweitzer et al. [12]

^b^Using split-group analysis of data from Schweitzer et al. [13]

^c^Item not included in research interview

### Psychiatric symptoms

Table 4 shows the mental health symptoms reported by participants, including means, standard deviations, and ranges for symptoms of traumatization, depression, anxiety, and somatization. Based on the application of HTQ and HSCL-37 suggested cut-offs [28], participants’ psychiatric distress met levels symptomatic for traumatization (41%), PTSD (20%), anxiety (29%), and depression (41%). Many women (42%) also reported high levels (≥ 1.75) of somatization. Table 4 also shows mental health symptoms reported by the Sudanese and Burmese groups [12, 13].

**Table 4:** Proportion of women-at-risk participants reporting mental health symptoms, with means, standard deviations, and ranges: comparison with Sudanese^a^ (*n* = 15) and Burmese^b^ (*n* = 34) women who arrived through Humanitarian (201) or Refugee (subclass 200) visas

Pathology	Women-at-risk			Sudanese^a^			Burmese^b^		
	Mean	SD (range)	%	Mean	SD (range)	%	Mean	SD (range)	%
Trauma^c^	1.89	0.61 (1–3.4)	41^e^	1.74	0.59 (1–3.1)	27^e^	1.78	0.66 (1–3.6)	25^e^
PTSD^c,d^	1.89	0.61 (1–3.4)	20^f^	1.74	0.59 (1–3.1)	13^f^	1.78	0.66 (1–3.6)	13^f^
Anxiety	1.55	0.60 (1–3.9)	29^g^	1.63	0.64 (1–3.4)	33^g^	1.39	0.34 (1–2.3)	21^g^
Depression	1.72	0.58 (1–3.5)	41^g^	1.69	0.56 (1–3.1)	33^g^	1.72	0.56 (1–3.3)	41^g^
Somatic	1.66	0.53 (1–3.6)	42^h^	1.57	0.54 (1–3.3)	20^h^	1.64	0.51 (1–2.8)	38^h^

^a^Using split-group analysis of data from Schweitzer et al. [12]

^b^Using split-group analysis of data from Schweitzer et al. [13]

^c^*n* = 32 for trauma symptoms and post-traumatic stress disorder

^d^Post-traumatic stress disorder

^e^Percentage of participants with HTQ symptom scores ≥ 2.0 (symptomatic range)

^f^Percentage of participants with HTQ symptom scores > 2.5 (indicates meeting PTSD diagnostic criteria)

^g^Percentage of participants with HSCL subscale scores ≥ 1.75 (i.e., symptomatic range)

^h^Percentage of participants with HSCL subscale scores ≥ 1.75

### Predictors of psychiatric symptoms

Table 5 provides a summary of regression analyses. For each dependent variable (symptoms of trauma, anxiety, depression, and somatization), individual factors were entered at Step 1, including age, region of birth, religion (Muslim vs. Christian), number of children (none vs. one or more), education completed (none or primary vs. secondary or tertiary), marital status (married or de facto vs. single, separated, or widowed), and English language skills (none or great difficulty vs. some difficulty or fluent). The number of trauma events experienced and level of post-migration living difficulties were entered at Step 2.

**Table 5:** Summary of Hierarchical Multiple Regression analyses predicting trauma, anxiety, depression, and somatic symptom scores for women-at-risk (*N* = 104)

Predictors		Trauma	Anxiety	Depression	Somatic
		*β*	*β*	*β*	*Β*
Step 1	Age, years	− 0.23*	− 0.25*	− 0.03	− 0.18
	Region of birth^a^	− 0.18	− 0.35**	− 0.26*	− 0.21
	Religion^b^	0.10	− 0.04	− 0.01	0.12
	Marital status^c^	− 0.12	0.10	0.12	0.07
	Children^d^	0.29**	0.27*	0.23	0.32*
	Education^e^	0.02	− 0.16	− 0.04	− 0.01
	English skills^f^	0.04	0.23*	0.18	0.20
Step 2	Trauma Events	0.42***	0.17	0.25*	0.26*
	PMLD	0.25**	0.17	0.23*	0.22*

*p < 0.05. **p < 0.01. ***p < 0.001

^a^Africa vs. other regions

^b^Muslim vs. other religions

^c^Married/de facto vs. single/separated/widowed

^d^No children vs. one or more

^e^Education completed (none/primary) vs. secondary/tertiary

^f^None/great difficulty vs. some difficulty/fluent

#### Trauma symptoms

The overall model, including demographic variables, number of trauma events, and level of post-migration living difficulties, was significant (*F* (9, 94) = 6.83, *p* < 0.001), accounting for 39.5% (adjusted *R*^*2*^ = 33.7%) of trauma symptom variance. Demographic variables accounted for 11.9% (*p* = 0.09) of trauma symptom variance. Trauma events experienced and level of post-migration living difficulties together explained an additional 27.6% (*p* < 0.001) of trauma symptom variance after accounting for effects of demographic variables.

Number of trauma events experienced, having children, level of post-traumatic living difficulties, and age (in order of importance) each made statistically significant unique contributions to trauma symptoms after controlling for overlapping effects of all other independent variables.

Higher trauma symptomatology was associated with higher number of trauma events and higher level of post-migration living difficulties. Follow-up analyses revealed that participants with children had higher trauma symptom scores (*M* = 2.01) than participants without children (*M* = 1.72). Trauma symptoms were higher in women aged 18–30 years (*n* = 51, *M* = 1.92) and 31–40 years (*n* = 26, *M* = 1.95) than in women aged 41–50 years (*n* = 22; *M* = 1.66). While trauma scores were highest for women aged 51–60 years (*n* = 3, *M* = 2.75) followed by women 61–70 years (*n* = 2, *M* = 1.88), low subsamples in these two groups caution inference. When including the four most prevalent PMLD items (as shown in Table 3) into the regression models, only ‘loneliness and boredom’ was significantly associated with higher trauma symptomatology (*β* = 0.24; *p =* 0.02).

#### Anxiety symptoms

The overall model, including demographic variables, number of trauma events, and level of post-migration living difficulties, was significant (*F* (9, 94) = 3.63, *p* = 0.001), accounting for 25.8% (adjusted *R*^*2*^ = 18.7%) of anxiety symptom variance. Demographic variables accounted for 19.2% (*p* = .004) of anxiety symptom variance. Trauma events experienced and level of post-migration living difficulties together explained an additional 6.6% (*p* = 0.018) of anxiety symptom variance after accounting for the effects of demographic variables.

Region of birth, having children, age, and English language skills (in order of importance) each made statistically significant unique contributions to anxiety symptoms after controlling for the overlapping effects of all other independent variables.

Follow-up analysis revealed that participants from regions other than Africa had higher anxiety symptom scores (*M* = 1.78) compared to those from Africa (*M* = 1.49). However, additional analyses according to region of birth revealed that, while women from Afghanistan (*n* = 12, *M* = 2.02) and South-East Asia (*n* = 3, *M* = 2.03) did have higher anxiety scores than women from Africa, anxiety scores of women from West Asia (Iran, Iraq, and Syria; *n* = 7, *M* = 1.27) were in fact lower than reported by African women. Nevertheless, the low subsamples in some groups caution inference.

Follow-up analyses also revealed that participants with children had greater anxiety symptoms (*M* = 1.65) than those without children (*M* = 1.40). While low subsamples in some groups caution inference, women aged 51–60 years had the highest mean anxiety score (*n* = 3, *M* = 2.33), followed by women aged 31–40 years (*n* = 26, *M* = 1.74), 18–30 years (*n* = 51, *M* = 1.54), 41–50 years (*n* = 22, *M* = 1.29), and 61–70 years (*n* = 2, *M* = 1.05). Participants who reported fluent English or some difficulty with English skills had greater anxiety symptom scores (*M* = 1.63) than those who reported great difficulty or no English skills (*M* = 1.50). None of the four most prevalent PMLD items was significantly associated with anxiety symptoms.

#### Depression symptoms

The overall model, including demographic variables, number of trauma events, and level of post-migration living difficulties, was significant (*F* (9, 94) = 4.28, *p* < 0.001), accounting for 29.1% (adjusted *R*^*2*^ = 22.3%) of depression symptom variance. Demographic variables accounted for 15.1% (*p* = 0.024) of depression symptom variance. Trauma events experienced and level of post-migration living difficulties together explained an additional 13.9% (*p* < 0.001) of depression symptom variance after controlling for the effects of demographic variables.

Region of birth, number of trauma events experienced, and level of post-migration living difficulties (in order of importance) each made statistically significant unique contributions to depression symptoms after controlling for the overlapping effects of all other independent variables.

Follow-up analyses revealed that participants from regions other than Africa had higher depression symptom scores (*M* = 1.88) than those from Africa (*M* = 1.68). Additional analyses according to region of birth revealed that depression scores of women from Afghanistan (*n* = 12, *M* = 2.07) and South-East Asia (*n* = 3, *M* = 2.12) were higher than those of women from Africa. Women from West Asia (*n* = 7, *M* = 1.46) had lower depression than women from Africa. Low subsamples caution inference regarding some region-of-birth groups. Higher depression symptomatology was associated with greater number of trauma events experienced and greater level of post-migration living difficulties. Of the four most prevalent PMLD items, only ‘loneliness and boredom’ was significantly associated with higher depression symptomatology (*β* = 0.38; *p =* 0.001).

#### Somatic symptoms

The overall model, including demographic variables, number of trauma events, and level of post-migration living difficulties, was significant (*F* (9, 94) = 4.29, *p* < 0.001), accounting for 29.1% (adjusted *R*^*2*^ = 22.3%) of somatic symptom variance. Demographic variables accounted for 15.6% (*p* = 0.02) of somatic symptom variance. Trauma events experienced and level of post-migration living difficulties together explained an additional 13.5% (*p* < 0.001) of somatic symptom variance after controlling for the effects of demographic variables.

Number of children, number of trauma events experienced, and level of post-migration living difficulties (in order of importance) each made statistically significant unique contributions to somatic symptoms after controlling for the overlapping effects of all other independent variables.

Follow-up analysis revealed that participants with children had higher somatic symptom scores (*M* = 1.77) than those without children (*M* = 1.50). Higher somatic symptomatology was associated with a greater number of trauma events and higher level of post-migration living difficulties experienced. ‘Loneliness and boredom’ was significantly associated with higher somatic symptomatology (*β* = 0.25; *p =* 0.03).

## Discussion

This research aimed to determine the prevalence of psychiatric symptoms (trauma, anxiety, depression, and somatic) in a sample of refugee women-at-risk recently resettled in Australia and to index the women’s experience of traumatic events and post-migration difficulties. The research also aimed to determine individual, trauma, and post-migration factors contributing to the women’s psychiatric symptoms.

### Traumatic experiences

This research documented women’s experience of multiple and severe traumatic events. Of note, nearly two-thirds of women had lived without basic necessities of life, including food, water, and shelter, or had witnessed extreme violence, such as the murder of family, friends, or strangers. Over one-third had endured rape or sexual abuse. The women’s experience of trauma aligns with reports by other refugee groups [8]. However, the proportion of women-at-risk who had experienced serious injury, imprisonment or detention, being lost or kidnapped, and rape or sexual abuse, was over double that reported by women resettled through other humanitarian and refugee visas in previous research [12, 13]. The high proportion of women-at-risk reporting these traumatic events likely reflects the women’s unique vulnerabilities and limited autonomy across their refugee journeys and, given a proposed dose–response effect [10], speaks of the importance of mental health assessment and monitoring for women in this group.

### Post-migration living difficulties

The post-migration difficulties of most concern to the women were relational in character, with over two-thirds revealing worry about family overseas, over one-third reporting problems with communication, and nearly one-third reporting difficulties with loneliness and boredom. Findings are consistent with Gonsalves’ [30] stage of early arrival (1 week to 6 months), whereby newly arrived refugees remain ensconced in their own cultural frameworks, with their most important tasks being to “*learn their surroundings, remain involved with homeland, and meet fellow refugees*” (p. 385). A high proportion of women in the Sudanese and Burmese groups also reported worry about family overseas and communication as serious concerns [12, 13]. It is perhaps unsurprising that connecting with family in worrying circumstances overseas is a serious concern for many, given that the loss of home and family can give rise to significant distress [31], and learning to communicate in a new country is a key survival task.

Few women-at-risk reported experiencing serious difficulties with racial discrimination, adjusting to cultural life in Australia, or employment. Having been resettled for less than 3 months on average, it is possible that refugee women-at-risk were yet to experience what Gonsalves [30] termed a period of destabilization (from 6 months to 3 years after arrival), during which one of the main tasks of refugees is to acquire survival tools such as employment. It is interesting to note that 57% of women in the Sudanese group, who had been resettled for just under 2 years on average, endorsed employment as a serious problem.

The high proportion of women-at-risk reporting loneliness and boredom as serious problems suggest that the current relationship needs of many women are unmet. In previous qualitative research, Somali refugee women described significant emotional distress from “*an overriding sense that social networks have been eroded and fractured*” ([20], p. 96). This is likely to be even more so for women-at-risk who arrive without spouses. Although many of the women likely maintain an interdependent self-construal, defining themselves in relation to their communities, relationships with like-ethnic community members can be complex; indeed, women-at-risk report being stigmatized and excluded by community members or excluding themselves to avoid exploitation [17, 18]. Considering social participation has been found to have a protective effect for migrants, particularly for those experiencing discrimination [32], findings point to the significance of fostering connections of newly arrived women-at-risk with family overseas, with other women-at-risk, and with host-community members.

### Psychiatric symptoms

The high number of traumatic events and level of post-migration living difficulties experienced by women-at-risk have the potential to underpin increased vulnerability to psychiatric distress [10, 13]. Significant proportions of women reported trauma (41%), anxiety (29%), and depression (41%) at symptomatic levels, with many reporting substantial symptoms of somatization (42%) or exceeding the threshold for PTSD (20%). Proportions are substantially higher than refugee prevalence rates for PTSD (9%) and major depressive disorder (5%) reported in meta-analytic research [33], and are also higher than data derived from a reference group of women who entered Australia under the regular Refugee Humanitarian Program. The Program helps people subject to persecution or substantial discrimination in their home countries who do not meet the criteria for the women-at-risk program.

Since gender has been previously associated with poorer refugee mental health [12, 23], the symptom profile of women-at-risk requires examination within the context of women resettled through other visa categories. While we recognize that the small sample sizes and differences in participant characteristics of the Sudanese and Burmese data presented [12, 13] preclude controlled empirical comparison with the current sample of women-at-risk, the mean trauma symptom score, the proportion of participants symptomatic for trauma, and the proportion of participants meeting the PTSD cutoff were notably higher for women-at-risk than for women who had entered Australia on other visas. Women-at-risk appeared to have similar levels of depression and somatization to Burmese women resettled in Australia for a similar period, but appeared to have higher anxiety. Unlike women from Sudanese and Burmese comparison groups, women-at-risk are without male family support. Further, based on qualitative research [17], women-at-risk are subject to exclusion from their own ethnic communities, and therefore may lack important resources to mitigate poor mental health.

### Contribution of individual, trauma, and post-migration factors to psychiatric symptoms

The number of trauma events experienced by women was the most important predictor of trauma symptoms, with a higher number of trauma events predicting a higher trauma symptomatology, consistent with hypotheses, and consistent with previous research findings of a dose–response relationship between the number of trauma events experienced and level of traumatization [10]. Interestingly, having children also predicted higher trauma symptoms, which may be understood in terms of an additional sense of vulnerability in being a single woman responsible and fearing for her children’s welfare but with limited resources. Consistent with previous research with Burmese refugees in Australia [13], higher levels of post-migration living difficulties predicted higher trauma symptoms. Contrary to our hypotheses and meta-analytic research with the wider refugee population that found being older was associated with poorer mental health [23], women-at-risk aged 18–40 years had higher trauma scores than women aged 41–50 years.

Neither trauma events experienced nor level of post-migration living difficulties predicted anxiety symptoms. Instead, region of birth was the most important predictor of anxiety. Women from Afghanistan and South-East Asia (Myanmar and Thailand) had higher anxiety scores than women from Africa (including Eritrea, Democratic Republic of Congo, Ethiopia, Sudan, South Sudan, Rwanda, Burundi, and Kenya), who had higher anxiety than women from West Asia (Iran, Iraq, and Syria). The findings concur with a meta-analytic review that found that region of birth predicts mental health outcomes [23]. People from the different regions may differ in levels or types of adversity experienced throughout their refugee journeys. When responding to symptoms, there is also a need to acknowledge different cultural dimensions and the meaning of symptoms for each person [34]. Having children also predicted higher anxiety symptoms, perhaps reflecting the concerns, practical tasks, and financial challenges of raising children in an unfamiliar culture with limited resources. Inconsistent with hypotheses and previous meta-analyses [23], women aged 18–40 years had higher anxiety scores than women aged 41–50 years. Interestingly, having greater English fluency predicted higher anxiety symptoms. Previous research with South-East Asian refugees in Canada found that English ability predicted depression in the longer term, but not during the initial period of resettlement [24]. Better language ability may be associated with increased expectations of gaining entry to training programs or employment, which may be experienced as additional demands, particularly when there is discrepancy between aspirations and employment outcomes.

Region of birth was the most important predictor of depression symptoms. Although the depression score was lower for African women than for women from other regions combined, closer examination revealed the depression score for West Asian women (Iran, Iraq, and Syria) was lower than that for African women. Consistent with hypotheses and previous research findings [9, 12, 13], a higher number of trauma events and higher levels of post-migration living difficulties predicted higher depression levels.

Having children was the most important predictor of higher somatic symptom levels. When considering the impact of the challenges that women-at-risk face in parenting with little support, attention to the cultural dimensions of psychological disorder may be useful, such as the ways in which different cultural groups might varyingly experience, conceptualize, and respond to phenomena [34]. Consistent with hypotheses and previous research findings [9, 12, 13], a higher number of trauma events experienced and higher levels of post-migration difficulties also predicted higher somatic symptom levels.

‘Loneliness and boredom’ was an important post-migration living difficulty that predicted trauma, depression, and somatic symptoms. This highlights the need to break down social isolation among refugee women-at-risk through women’s support groups and the creation of safe spaces for women to develop their networks, as well as providing psychosocial support and English language programs [17].

Nevertheless, the results need to be considered in the context of the research limitations. Caution is required in generalizing findings from this relatively small, non-random, heterogeneous sample of volunteers (with potential self-selection bias), resettled in a specific geographical region. For some comparisons, groups were small. Assessment of mental health symptoms relied on questionnaire responses. Although the cross-sectional design of the current research provided a pragmatic approach, it does not determine causality. Because participants had varied linguistic backgrounds, the research relied on interpreters, rather than back translation methods, raising potential for differences in survey administration. Although hierarchical regression models utilizing specified individual factors, trauma events, and post-migration living difficulties as predictors explained respectable proportions of symptom variance (approximately 26–40% depending on symptom category), substantial proportions of symptom variance (60–74%) remained unexplained. Future research, using additional predictor variables and a larger sample, could compare psychiatric symptoms of women-at-risk with women resettled on other visa categories controlling for demographic characteristics, and could also investigate differences in women-at-risk from different countries. Additionally, consideration may be given to extending the assessment to include positive wellbeing as conceptualized in terms of post-trauma growth. Relationships between the HTQ and HSCL questionnaire responses and full clinical assessment of each participant could also be examined.

## Conclusions

Developing an understanding of the factors that significantly impact the mental health of resettled women-at-risk benefits mental health practice and holds particular worth in guiding resettlement policies and programs. This research highlights the vulnerability of refugee women-at-risk to traumatic events during their refugee journeys, their post-migration struggles, and the impact of these experiences on their mental health, reflected in the women’s high levels of trauma, anxiety, depression, and somatic symptoms. Research findings augment previous qualitative research that identified major service gaps for this vulnerable group of women, including need for increased financial support, improved pre-arrival information to prepare women coming to Australia, specialist women’s health services, and secure accommodation for women upon their initial arrival in Australia [17]. Results from the current research also underline the importance of recognizing both the pre- and post-migration experiences of women-at-risk, remaining mindful of demographic risk factors, and assessing the women for potential psychiatric symptoms early in resettlement to distinguish women in need of intervention. Overall, research findings contribute to the very limited empirical literature on refugee women-at-risk, providing initial benchmarks for this group. Findings also contribute to the broader emerging literature investigating the mental health of refugees to inform mental health assessment, practice, and policy.

## Abbreviations

HSCL: Hopkins Symptom Checklist
HTQ: Harvard Trauma Questionnaire
PMLD: Post-migration Living Difficulties Checklist
PTSD: post-traumatic stress disorder
UNHCR: United Nations High Commissioner for Refugees
